# Characterization of Longitudinal Testosterone, Cortisol, and Musth in Male Asian Elephants (*Elephas maximus*), Effects of Aging, and Adrenal Responses to Social Changes and Health Events

**DOI:** 10.3390/ani12101332

**Published:** 2022-05-23

**Authors:** Sharon S. Glaeser, Katie L. Edwards, Stephen Paris, Candace Scarlata, Bob Lee, Nadja Wielebnowski, Shawn Finnell, Chaleamchat Somgird, Janine L. Brown

**Affiliations:** 1 Oregon Zoo, 4001 SW Canyon Rd., Portland, OR 97221, USA; candace.scarlata@oregonzoo.org (C.S.); blee@cabq.gov (B.L.); nadja.wielebnowski@oregonzoo.org (N.W.); sfinnell@white-oak.org (S.F.); 2 North of England Zoological Society, Chester Zoo, Upton-by-Chester, Chester CH2 1EU, UK; k.edwards@chesterzoo.org; 3 Center for Species Survival, Smithsonian Conservation Biology Institute, Smithsonian National Zoological Park, Front Royal, VA 22630, USA; pariss@si.edu (S.P.); brownjan@si.edu (J.L.B.); 4 ABQ BioPark, 903 10th St. SW, Albuquerque, NM 87102, USA; 5 White Oak Conservation, 581705 White Oak Road, Yulee, FL 32097, USA; 6 Department of Companion Animal and Wildlife Clinics, Faculty of Veterinary Medicine, Chiang Mai University, Chiang Mai 50100, Thailand; chaleamchat.s@cmu.ac.th; 7 Center of Elephant and Wildlife Research, Faculty of Veterinary Medicine, Chiang Mai University, Chiang Mai 50100, Thailand

**Keywords:** elephant, musth, testosterone, cortisol, glucocorticoids, adrenal activity, reproduction, aging, health, welfare

## Abstract

**Simple Summary:**

The conservation of endangered species and sustainability of managed populations requires good health and welfare of individuals. Male elephants experience a biological phenomenon called “musth”, characterized by a suite of physiological and behavioral changes which serve to facilitate copulation between the sexes, but can also pose unique challenges under human care. This study characterized longitudinal patterns of circulating testosterone and cortisol in relation to musth in four adult Asian elephant bulls and assessed age-related effects on musth activity and adrenal responses to social changes and clinical health events. All bulls exhibited regular annual musth cycles, and there was no clear evidence of chronically elevated cortisol secretion outside of musth. Circulating cortisol covaried positively with testosterone and musth, highlighting intrinsic patterns that should be considered when evaluating the impact of changes on adrenal glucocorticoid activity. Testosterone decreased with age in sexually mature bulls, whereas age-related changes in cortisol varied across individuals, with the three older bulls showing the greatest rate of change during musth. Changes in cortisol were associated with treatment for *Mycobacterium tuberculosis* in two bulls, but not with activation of disease. In contrast to physiological factors, there was no evidence that social changes involving male herdmates impacted adrenal glucocorticoid activity in the short term. This study highlights the importance of longitudinal hormone monitoring to track changes in physiological function and identify factors that may influence welfare, which is important for making more informed decisions on how to manage male elephants under varying degrees of human care.

**Abstract:**

The conservation of endangered species and sustainability of managed populations requires considerations to ensure the health and welfare of individuals. Male elephants experience a biological phenomenon called “musth”, which is characterized by increased testosterone production, temporal gland secretion and urine dribbling, heightened aggression and sexual behavior, and therefore can pose unique challenges for human safety and animal welfare. This study characterized longitudinal (9 to 22 years) patterns of circulating testosterone and cortisol in relation to musth in four adult Asian elephant bulls spanning ages from 12 to 54 years. Age-related effects on musth activity and adrenal responses to social changes and clinical health events were also examined. All bulls exhibited regular annual musth cycles. Circulating cortisol covaried positively with testosterone and musth, highlighting intrinsic patterns that should be considered when evaluating the impact of social, health, and environmental changes on adrenal glucocorticoid activity. Except for an end-of-life cortisol increase in one bull, there was no clear evidence of chronically elevated cortisol secretion outside of musth in any individual. Testosterone decreased with age in sexually mature bulls, whereas age-related changes in cortisol varied across individuals, with the three older bulls showing the greatest rate of change during musth versus inter-musth periods. In contrast to physiological factors, there was no evidence of social factors, such as addition of a new male and death of male herdmates, impacting adrenal glucocorticoid activity in these bulls in the short term. Changes in cortisol were associated with treatment for *Mycobacterium tuberculosis* (*M. tb*) in two bulls, increasing after start of treatment and decreasing with cessation of treatment, but were not clearly associated with activation of disease. This study highlights the importance of longitudinal hormone monitoring to track changes in physiological function and responses to social, health, and environmental change in elephant bulls, which is important for making more informed decisions on how to manage male elephants under varying degrees of human care to ensure welfare and safety.

## 1. Introduction

Musth is a physiological and behavioral phenomenon characterized by increased testosterone, heightened aggression or unpredictability, increased sexual behavior, and a temporary rise in dominance in both wild and captive Asian [1,2,3,4,5] and African [1,6,7,8,9,10] elephant bulls. Bulls advertise musth status through a variety of chemicals exuded in temporal gland secretions and urine [5,11,12,13], and secretions from the paired facial temporal glands and dribbling of urine from the penile prepuce characterize physical signs of musth [3,5,6,8,9,11,12,14,15] which are also visible. Previous studies of musth in captive Asian bulls showed an onset of temporal gland secretions, and urine dribbling occurred as testosterone began to increase, or within a few weeks thereafter [4,16]. Nutrition is an important factor in the expression of musth, and in both wild and captive elephants, musth generally terminates with a significant decline in body condition [7,14]. Musth in Asian bull elephants normally occurs annually or biannually during approximately the same time each year for an individual, and varies among bulls in both duration and months of occurrence [3]. However, instances of irregular or near continuous musth-like symptoms have been observed in zoo-housed bulls, perhaps due to lack of social suppression by other bulls or due to a consistently high level of nutrition [16]. From a management perspective, because aggression often is associated with musth, it can create human safety and animal welfare concerns under certain circumstances. It is therefore important to better understand the physiological changes associated with musth, the factors influencing the expression of musth, and to have tools available to properly assess the musth condition and the well-being of male elephants of all ages.

Measures of glucocorticoids (GCs) are important for understanding how individuals respond to challenges or changes in their environment. GCs are secreted from the adrenal cortex in response to stimuli, moderating subsequent physiological processes, both beneficial and detrimental [17,18,19,20]. The primary role of GCs is basic energy regulation; however, under stressful conditions, they facilitate physiological changes and are important mediators of coping [21]. In zoo settings, increased GCs in elephants have been observed in response to the opening of a new zoo [22], during flooring renovations [23], and introduction of an unfamiliar conspecific [24,25,26]. While not reproductive hormones per se, GC elevations in elephants are also associated with normal physiological states such as musth [16,27,28], the follicular phase of the estrous cycle [29,30], and pregnancy and parturition [29,31,32]. In males of other species, GCs increase during rut and are associated with mating, aggressive behavior, and dominance [33,34,35,36,37,38].

In long-lived social species such as elephants, age-related and social factors are also important considerations for species management. It is now recognized that wild bull elephants have complex social structures and relationships [39,40,41]. Hartley et al. [42] reports relatively low rates of musth expression in elephant bulls in European and North American zoos, perhaps because the vast majority are under 20 years of age. Furthermore, many of those bulls have restricted opportunities for social interactions, either with females or other bulls. Ganswindt et al. [43] hypothesized that for wild African elephants, associations with non-musth bulls may have a social buffering effect on musth bulls based on the finding that concentrations of fecal GCs were lower in musth bulls in the presence of non-musth bulls as compared to when musth bulls were alone. So, perhaps the housing of multiple bulls is beneficial to each as they experience musth, whether they are in physical contact or not. Olfactory and acoustic communication are critical for mediating interaction among wild elephant groups [44] and represent the primary forms of communication among bulls in zoos as they are rarely kept together [42].

This study involved longitudinal assessments (from 9 to 22 years) of serum testosterone and cortisol concentrations in four male Asian elephants at a North American zoo with a long history of continuous hormone monitoring. The age range spanned from 12 to 54 years, and each male exhibited regular annual musth. Three bulls developed and were treated for tuberculosis (TB) during the study. This extensive dataset allowed us to examine variation in hormone concentrations in relation to musth, age, TB development and treatment, and health declines leading to death. We also examined adrenal response to social changes after a new bull was transferred to the zoo and after the deaths of male conspecifics. Outcomes of this study can help us gain a better understanding of underlying conditions affecting patterns in testicular and adrenal GC activity, and how physiological or environmental changes may affect the health and welfare of male Asian elephants.

## 2. Materials and Methods

### 2.1. Animals and Sample Collection

Subjects were adult male Asian elephants (*Elephas maximus indicus*) (*n* = 4) housed at the AZA-accredited Oregon Zoo. Elephants were both wild-born (*n* = 2) and zoo-born (*n* = 2), with an age ranging from 12 to ~54 years during the study (Table 1). This study was approved by the Animal Welfare Committee and the Research Review Committees of the Oregon Zoo (OZ).

At any given time during the study period, two to three adult males were rotated among two outdoor yards and five indoor enclosures. All elephants had olfactory and auditory contact, but these bulls did not share enclosures and had no physical contact with each other, although they had some visual contact through barriers. All bulls in this study had at least occasional physical contact with females for socialization and/or breeding. The three oldest bulls sired multiple offspring through natural breeding. Access to females was not limited to either musth or non-musth periods, but rather to behavioral signs. If any elephant, male or female, exhibited behavior suggesting they preferred to be separated (e.g., standing at an access door away from other elephants, repeatedly moving away), then keepers opened access doors to allow them to do so. Male1 was given access to females only for breeding. Male2 was given access to the female herd for breeding and socialization, but unless a female was in estrus, he would choose to separate from the females. Male3 was well-integrated with the females and male offspring, and regularly spent time with them for socialization as well as for breeding. Male4 spent time with a compatible female, but not with the entire group. All bulls were managed in a protected-contact system, whereby keepers and animals did not share the same space.

Social hierarchy among the bulls at any given time was determined by observations of behavioral posturing (bull assuming a dominant, neutral, or subordinate posture) and the physical location in adjacent yards in reaction to visual contact with another bull (Mike Keele and Bob Lee, pers comms). Over the course of the study, there were two triads and two dyads of adult bulls: Male1–Male2–Male4 (1994–2003), Male2–Male4 (2003–2005), Male2–Male3–Male4 (2005–2015), and Male2–Male3 (2015). Male4 was subordinate in all groups. Male1 ranked higher than Male2, but later in the study, Male2 imposed dominance and was perceived as being higher in rank. Rank was not clear between Male2 and Male3 as both were large, dominant bulls; however, Male2 was aging, and they were not given access to each other to assess dominance. As is typical in male elephants, any non-musth bull deferred dominance to a musth bull.

Elephants were trained for voluntary blood collection (without sedation) as part of routine management. Blood was collected (3–9 mL) in red-top serum separator tubes from an ear or leg vein by elephant care staff. Blood was maintained at approximately −4 °C, and then within a few hours it was centrifuged at 1500× *g* to separate serum and stored at −20 °C or colder until analysis. Samples were collected weekly, and in the morning to control for diurnal patterns of hormone secretion [45]. Serum testosterone was measured weekly for routine management starting in 2006. Prior to 2006, serum testosterone was measured as part of research projects [5,12,13,16,46]. Serum cortisol was measured in all stored samples for this research project and all samples were processed over a period of 3 months in 2017.

### 2.2. Hormone Analysis

Testosterone concentrations in unextracted serum were initially measured using a solid-phase ^125^I testosterone radioimmunoassay (RIA) (Siemens Healthcare Diagnostics Inc.) following the methods of Brown et al. [16]. Samples above high standard (below 20% binding) were diluted in the zero calibrator (or standard) and re-analyzed. Assay sensitivity was 0.10 ng/mL. The RIA antibody cross-reacted with testosterone (100%), 19-nortestosterone (22%), 4-estren-17-ol-3-one (20%), 11-ketotestosterone (16%), 5α-dihydrotestosterone (3.4%), 19- hydroxandrostenedione (2.0%), methyltestosterone (1.7%), 11β-hydroxytestosterone (1.2%), 4-estren-7α-methyl-17β-ol-3-one (1.1%), ethisterone (0.7%), androstenedione (0.5%), 5β-androstan-3α,17β-diol (0.4%), 5-androstan-3β,17β-diol (0.2%), 4-5(10)-estren-17α-ethinyl-17β-ol-3-one (0.2%), triamicinolone (0.2%), and norethindrone (0.1%).

This RIA was discontinued at the end of 2014, after which serum testosterone was quantified through a double-antibody testosterone enzyme immunoassay (EIA) using an anti-testosterone polyclonal primary antibody (R156/7; C. J. Munro, University of California, Davis, CA, USA) and testosterone–horseradish peroxidase (HRP) tracer (C. Munro, UC, Davis, CA, USA). Microtiter plates were coated following methods described in Edwards et al. [47] with the exception that wells were emptied and plates blotted dry to remove unbound antibody after the first incubation rather than washed with wash buffer. The R156/7 antibody (1:187,500) and HRP (1:100,000) were diluted in assay buffer (Cat. No. X065, 5X, Arbor Assays, Ann Arbor, MI, USA). On the day of analysis, a pre-coated goat anti-rabbit IgG plate was equilibrated to room temperature (~30 min). Unextracted serum was incubated with equal quantities of dissociation reagent (X017, Arbor Assays) for 5 min and then diluted in assay buffer (1:20 to 1:100) for analysis. Testosterone standards (0.023–6.0 ng/mL; Steraloids, Inc., Newport, Rhode Island, NY, USA), internal controls, and samples (50 µL each) were added to each well in duplicate, followed by 25 µL of HRP, then 25 µL of primary antibody to all wells except non-specific binding wells. The loaded plate was sealed and incubated at RT with shaking (500 rpm) for 2 h. Unbound components were removed by washing 3 times with wash solution (Cat. No. X007, 20X, Arbor Assays, Ann Arbor, MI, USA) and blotted dry, followed immediately by adding 100 µL of a chromogen solution containing high-kinetic tetramethylbenzidine (TMB) (2.5 mmol/L, Prod. No. TMB-HK, Moss, Inc., Pasadena, MD, USA) to each well. The plate was sealed again and incubated at RT without shaking for 30 min, and then the reaction was halted by adding stop solution (50 μL; 1N HCL, Ricca Chemical, Arlington, TX, USA). Optical densities were determined by a Dynex plate reader at 450 nm with a reference of 630 nm. Samples below 20% binding were re-analyzed at a higher dilution. Assay sensitivity (based on 90% binding) for the testosterone EIA was 0.015 ng/mL. The R156/7 antibody cross-reacted with testosterone (100%), 5α-dihydrotestosterone (57.37%), androstenedione (0.27%), and less than 0.10% for 17 other steroids tested (C. Munro).

Cortisol concentrations in unextracted serum samples were measured using a solid-phase cortisol ^125^I radioimmunoassay (RIA) (CortiCote, MP Biomedicals, Santa Ana, CA, USA; catalog # 06B256440) with modifications described in Edwards et al. [47]. Assay sensitivity was 2.5 ng/mL. Cross-reactivities for the cortisol RIA antibody were cortisol 100.0%, prednisolone 94.1%, 11-deoxycortisol 2.2%, prednisone 1.2%, corticosterone 1.2%, cortisone 0.8%, dexamethasone 0.8%, 17-hydroxyprogesterone <0.05%, and metyrapone <0.01%.

The RIAs were previously validated for elephants by Brown et al. [16] by demonstrating parallelism and >90% recovery of exogenous standard hormone, and with biological tests [16,48]. The testosterone EIA was also validated by demonstrating parallelism and >90% recovery of exogenous standard, and with biological tests of significant increases observed during musth. Testosterone concentration in samples measured using both the EIA and RIA were strongly correlated, *r*(49) = 0.93, although measured concentrations using the EIA were on average 1.8 times higher than the RIA. Thus, EIA results were divided by 1.8 for inclusion in this longitudinal dataset. The inter- and intra-assay coefficients of variation (CVs) were maintained below 15% and 10%, respectively, by re-analyzing samples or plates that fell outside these criteria.

### 2.3. Determination of Musth State

#### 2.3.1. Physical and Behavioral Signs of Musth

Physical and behavioral signs of musth were recorded by elephant care staff in daily keeper notes. Temporal gland secretion (TGS), urine dribbling (UD), and behavior changes were recorded using numerical scales defined by Scott [12].The Oregon Zoo collaborated on the development of a more detailed musth scale for the physical or visible signs of TGS (Table 2) and UD (Table 3), which was adopted from previous work by Jainudeen et al. [3], Poole [6], Scott [12], and Somgird et al. [15]. A single-page PDF of this musth scale is available upon request. Numerical record values were converted to this new scale for analysis (i.e., TGS = 1 changed to 2; TGS = 2 changed to 3). Table 4 shows the behavior changes that were typically associated with musth in this group of bulls. 

#### 2.3.2. Criteria for Defining Musth

The state of musth was defined by an increase in testosterone coincident with physical and/or behavioral signs of musth; stages of musth were differentiated by duration of increased testosterone and intensity of the physical and behavioral signs (Table 5). The minimum testosterone concentration for determining musth state was based on previous studies by Scott [12] and Brown et al. [16].

The pre- and post-musth stages were included in the full-musth state for analysis because musth occurs on a spectrum, and physiological changes (i.e., increased testosterone and volatile secretions) also occur in these transitional stages [3,13,15,46,49]. Furthermore, in some years the historical records indicate only that an animal was “in musth”, and the details of TGS, UD, and behavior were not included to help delineate pre- and post-musth from full-musth periods.

#### 2.3.3. Calculation of Individual Musth Thresholds

After musth periods were defined using the criteria in Table 5, testosterone concentrations corresponding to the beginning and end of musth were used to calculate minimum, median, and mean threshold concentrations for each individual. To aid future research, these thresholds were then compared to thresholds calculated using the methods of Chave et al. [27] to define musth based solely on testosterone concentrations.

### 2.4. Demographics and Social Life Events

Origin of birth, birth date, and life event data of births, deaths, and facility transfers (change in physical location) for each bull were obtained from the AZA Asian Elephant Regional Studbook [50]. During the study, one adult male was transferred to Oregon Zoo, and all four died, which constituted social life events (i.e., events involving herdmates), and events that individuals physically experienced (i.e., their own death).

### 2.5. Tuberculosis Diagnosis and Treatment

Three bulls in this study were diagnosed with *Mycobacterium tuberculosis* (*M. tb*), a primary causative agent of tuberculosis (TB). The outbreak diagnosis and treatment are documented in detail by Miller et al. [51]. The first case in 2013 (Male4) was detected by isolation of *M. tb* during routine trunk wash (TW) culture testing. Subsequent retrospective antibody analyses revealed seroconversion 1 year prior to diagnosis. Serological testing of all elephants identified two additional bulls (Male2 and Male3) with detectable antibodies, at which time these bulls remained culture-negative; subsequently *M. tb* was isolated from TW samples from these bulls (within 4 months for Male2 and 10 months for Male3). No clinical signs attributable to TB were detected in any elephant. All infected elephants received anti-TB therapy as described in detail by Miller et al. [51] (elephant A is Male4; B is Male2; C is Male3). Health records were reviewed to identify dates of the last negative and first positive TW to identify the period of transition to active shedding of *M. tb* (Male4: 14 months; Male2: 10 weeks; Male3: 9 weeks) and also anti-TB therapy start and end dates.

### 2.6. Health Declines

All four bulls died or were euthanized during the study. Male1 died of sepsis; no euthanasia was performed. Two bulls that were treated for TB were euthanized for reasons unrelated to *M. tb.* Male4 was euthanized due to chronic progressive arthritis in multiple joints, and was on anti-TB therapy for 20 months until the time of death. Male3 was euthanized for severe extensive chronic osteoarthritis, and was on anti-TB therapy for 15 months until time of death. Male2 was euthanized due to development of a drug-resistant strain of *M. tb*; his treatment was discontinued after confirmation of drug resistance, and he was euthanized 4 months later [51].

### 2.7. Data Analysis

Median, range, mean, standard deviation (SD), and coefficient of variation (CV) in serum testosterone and cortisol concentrations were calculated in Excel for each individual and all elephants combined for the entire study period, during musth and inter-musth periods, and in Male4, including the transition from a pre-pubertal to adolescent state and a first musth cycle. Differences between CVs were determined following methods in Glaeser et al. [29]. Significance was set at 0.05 for all analyses.

Generalized linear mixed models (GLMMs) were used following methods in Glaeser et al. [29] to investigate changes in mean testosterone concentrations in association with reproductive state (prepubertal, adolescent), musth state (musth, inter-musth), age, origin of birth, period of transition to active shedding of *M. tb*, start/end of anti-TB therapy, health decline leading to death or euthanasia, and in response to social life events (transfers and deaths of other bulls). GLMMs allow random effects to be incorporated into the model [52,53] to control for non-independence of data, which in this study involved repeated serum samples per subject. Kolmogorov–Smirnov normality tests and examination of plots showed distributions of hormone data were non-normal, so hormone data were log10-transformed to improve the distribution for the GLMMs.

Separate models were created for each elephant to investigate the effect of physiological and social changes on an individual basis, and with all elephants combined to investigate group-level effects of age and origin of birth. GLMMs were based on models by Edwards, et al. [54,55] and Glaeser et al. [29]. Reproductive stage (prepubertal, adolescent), musth state, transition to active shedding of *M. tb*, anti-TB therapy, social life events, health decline, and 10-year age categories were fitted individually as categorical fixed effects; age was fitted as a continuous fixed effect. Hormone data were the dependent variables in each model.

Event-based effects (social life events, TB transition, anti-TB therapy, and health decline leading to death or euthanasia) were defined with time periods surrounding the event. Social life events (transfer and death of other males) were classified as pre- and post-event, with an equal duration of 30 days before and 30 days after the event (total of 60 days), and with the timeframe extended to 60 days before and after the event to determine if the addition or removal of a bull would have an impact on adrenal GC activity over a longer time period. The period of transition to active shedding of *M. tb* was defined as time between the last culture-negative TW to detection of active *M. tb*, which differed across individuals. Comparisons were made between this transition period and the preceding time period of equal duration, in 30-day blocks, to locate any acute changes in adrenal GC activity associated with disease transition. The start and end of anti-TB therapy were classified with an equal duration of 30 days before and 30 days after therapy start or end (total 60 days). The period leading up to death by euthanasia (*n* = 3) and to natural death (*n* = 1) were modeled with a timeframe of the final 30 days of life compared to the preceding 30 days (total 60 days). The day the event occurred was included in the post-event time period. The models were first run to determine differences in hormone concentration with musth state, and then these effects were taken into account by inclusion as covariates in the event-based models.

## 3. Results

### 3.1. Longitudinal Serum Testosterone Patterns

Serum testosterone concentrations (median, range, and mean) and variability (SD and CV) overall and during musth and inter-musth periods are presented in Table 6. Mean hormone concentrations were highly variable within and among individual bulls during both musth and inter-musth periods.

#### 3.1.1. Musth Patterns

**Figure 1:**
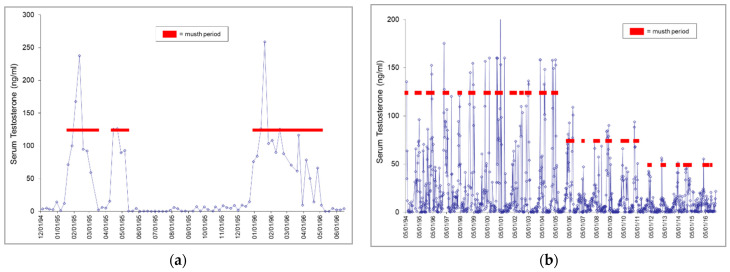

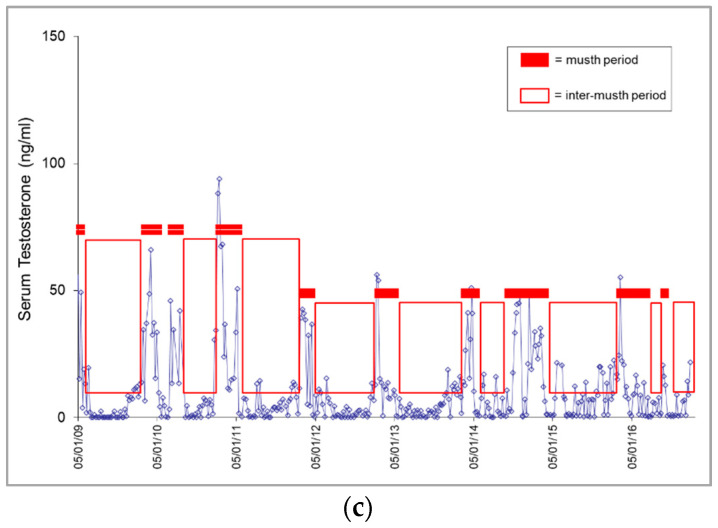
Patterns of testosterone during musth. (**a**) Male1: profile shows two annual musth cycles, with the first musth cycle split into two time periods occurring within the time frame of the second musth cycle; (**b**) Male2: profile shows declines in peak testosterone concentrations during musth with advancing age; (**c**) Male2: profile shows a greater percentage of time with increased testosterone during inter-musth periods from 2014 to end of life.

Musth was characterized by elevated serum testosterone concentrations corresponding to varying duration and intensity of temporal gland secretion (TGS), urine dribbling (UD), and behavioral changes. In general, behavior changes were noted prior to TGS and UD, and TGS and UD occurred as testosterone began to increase or within a few weeks thereafter. All bulls exhibited regular annual musth cycles, which generally occurred between late winter and mid-summer in the two oldest bulls (Male1 and Male2), followed by a cycle from mid-summer to late fall in the youngest bull (Male4), then from late summer to winter in the bull that transferred into the facility (Male3). Male1 exhibited annual musth over a 3-month range of musth start dates, and in some years exhibited musth in two distinct time periods within the time frame that in previous or subsequent years had comprised only one musth event (Figure 1a). There was no evidence of synchronization; however, in some years, musth cycles across individuals overlapped more than others.

There were no extended periods of elevated testosterone that were not associated with recorded behavioral and physical musth signs, with some exceptions. In the 1990s, the two oldest bulls sometimes exhibited a second shorter period of elevated testosterone after coming out of musth, with keeper records stating that an immediate return to full daily diets caused them to go back into musth. Feeding regimens were changed to increase hay and grain slowly in the weeks after musth, and by 2001 a recurrence of musth was rare. Male2 and Male4 exhibited testosterone elevations when other bulls were in musth, sometimes coincident with 1 to 2 weeks of TGS and UD. Male3 sometimes showed elevations in testosterone and single days of TGS/UD surrounding ovulation in three females, and/or when he had access to the females for breeding or socialization. Male2 showed a decline in peak testosterone concentrations during musth with advancing age, from age 44 in 2006 to end of life at age 54 in 2017 (Figure 1b). His musth episodes from 2014 to end of life, which coincided with anti-TB therapy, were described by elephant care staff as “erratic”, and were characterized by a less intense expression of annual musth, but a greater percentage of time with increased testosterone concentrations (Figure 1c) and/or expression of musth-like behaviors during inter-musth periods in which the criteria for full musth (Table 5) were not met.

#### 3.1.2. Musth Threshold Testosterone Concentrations

Testosterone concentrations corresponding to the beginning and end of musth, as defined by elevated testosterone coincident with physical and/or behavioral signs (Table 5), resulted in musth threshold testosterone concentrations for each individual (Table 7). Not surprisingly, the minimum testosterone concentrations associated with physical and/or behavioral for each individual were similar to previously published thresholds using these criteria [12,16]. However, mean and median threshold testosterone concentrations for these individuals were more similar to the concentrations for first musth point using the methods developed by Chave et al. [27] based solely on testosterone concentrations.

#### 3.1.3. Effects of Age and Dominance Status on Testosterone across Bulls

There were no definitive relationships between higher mean, median (Table 6), or musth threshold (Table 7) testosterone concentrations and age or dominance in this study. The older and dominant bulls in the Male1–Male2–Male4 triad had the highest testosterone concentrations, and concentrations for Male2 remained higher than Male4 throughout this study; however, this dominance-related pattern was not exhibited in the Male2–Male3–Male4 triad. Furthermore, this longitudinal dataset allowed comparisons across dominant bulls at similar chronological ages (Male1, ages ~34–43; Male2 and Male3, ages ~34–44), and confirmed an almost four-fold range in mean testosterone concentrations across bulls in the same age group and dominance rank.

#### 3.1.4. Effects of Age on Testosterone within Individuals

**Figure 2:**
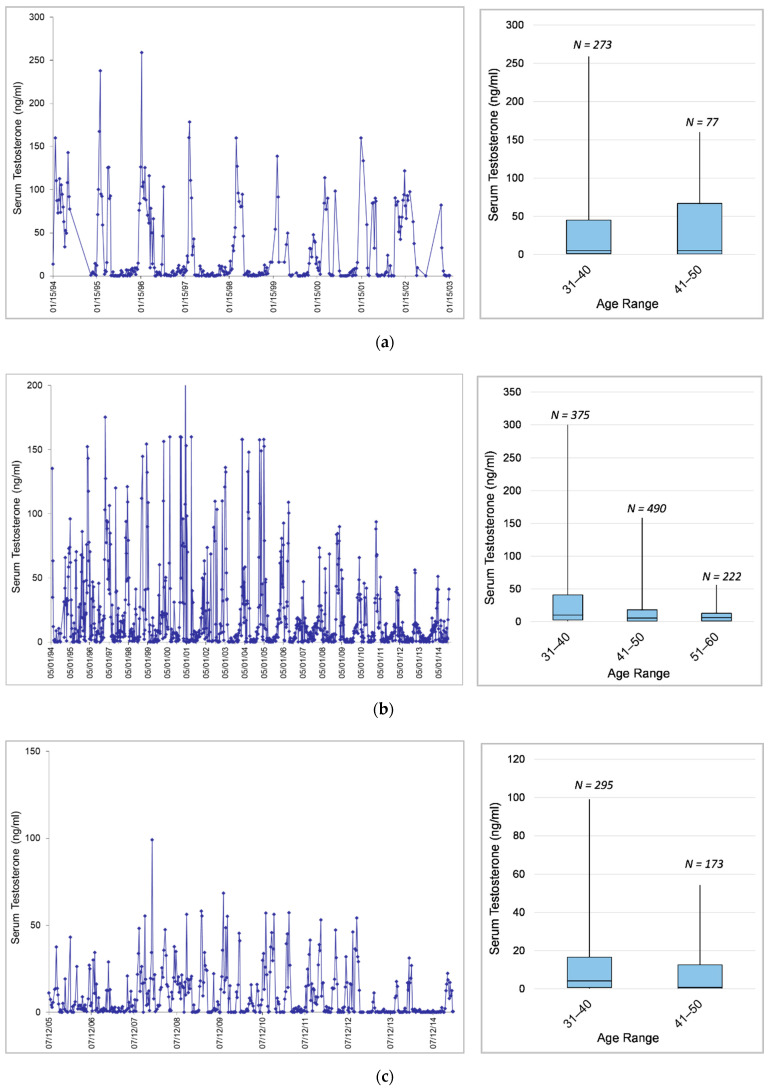

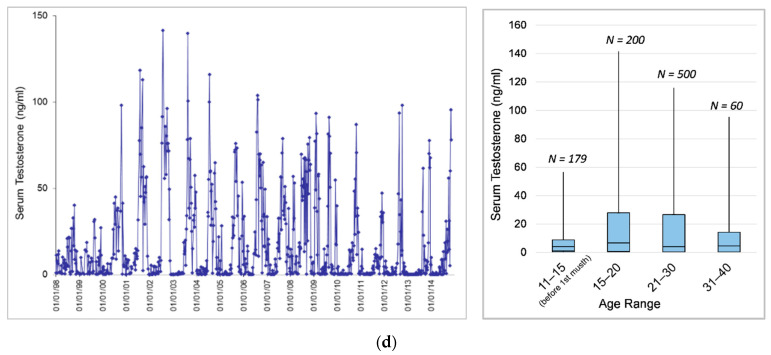
Changes in testosterone concentration in relation to age in individual bull elephants: testosterone profile (**left**), median and variability across age categories (**right**). (**a**) Male1; (**b**) Male2; (**c**) Male3; (**d**) Male4.

**Figure 3:**
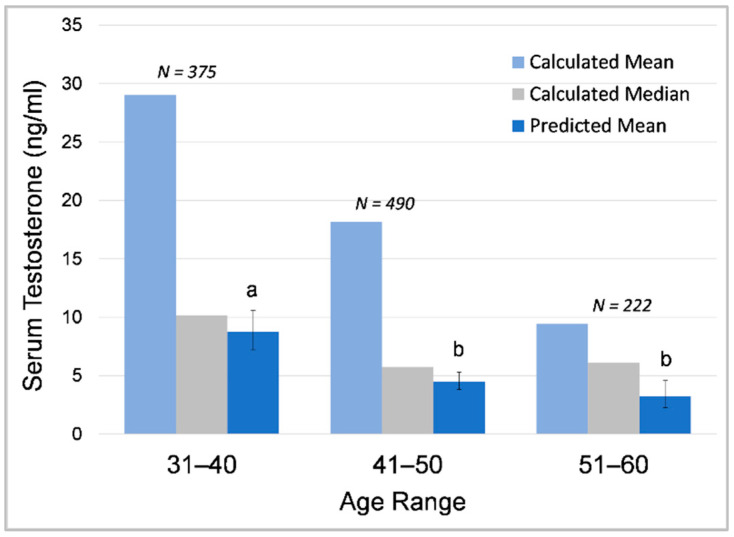
Changes in testosterone in relation to age in Male2, comparing calculated mean and median and GLMM-predicted mean. Different letters indicate significant differences in GLMM-predicted mean values between age categories.

Age was a significant predictor of serum testosterone concentrations, with testosterone decreasing with age overall in sexually mature bulls (Table 8). In Male2 and Male3, GLMMs predicted significantly lower testosterone in the older age categories; in Male1 and Male4, lower testosterone in older-age categories was also predicted (as indicated by negative effect size), but the difference between age categories was not significant. Furthermore, all bulls showed decreasing testosterone concentrations across age categories using comparisons of maximum and median testosterone concentrations in each age category and visual inspections of hormone profiles (Figure 2). Male1 (Figure 2a) showed a decrease in maximum concentrations in the older age category and an apparent increase in variability. Male2 (Figure 2b) and Male3 (Figure 2c) showed a decrease across age categories in maximum and median concentrations as well as variability. The youngest male, Male4 (Figure 2d), experienced his first musth cycle at 15 years of age and showed increasing testosterone with age as he entered puberty and matured during his early musth cycles until around age 20, and then exhibited decreasing testosterone. It is important to note that these age-related decreases occurred prior to detection and treatment of *M. tb* in all individuals, so *M. tb* was not a confounding factor in these age effects. Across age categories, GLMM predicted means of log10-transformed testosterone data were more similar to calculated median than mean values due to the data being extremely right-skewed (Figure 3).

### 3.2. Longitudinal Serum Cortisol Patterns

Serum cortisol concentrations (median, range, and mean) and variability (SD and CV) overall and during musth and inter-musth periods are presented in Table 9. Mean hormone concentrations varied within and among individual bulls during both musth and inter-musth periods.

#### 3.2.1. Effects of Testosterone, Musth, and Puberty on Cortisol

Cortisol was positively correlated with testosterone and was higher in musth compared to inter-musth periods in all individuals and all bulls combined (Table 10). Sexual maturation was a significant predictor of mean cortisol concentration (GLMM coefficient = 0.131, SE = 0.027, χ^2^ = 24.525, df = 1, *p* ≤ 0.001) in the bull that began exhibiting musth during the study (Male4), with cortisol being higher after sexual maturity compared to the pre-pubertal period.

#### 3.2.2. Effects of Age on Cortisol

**Figure 4:**
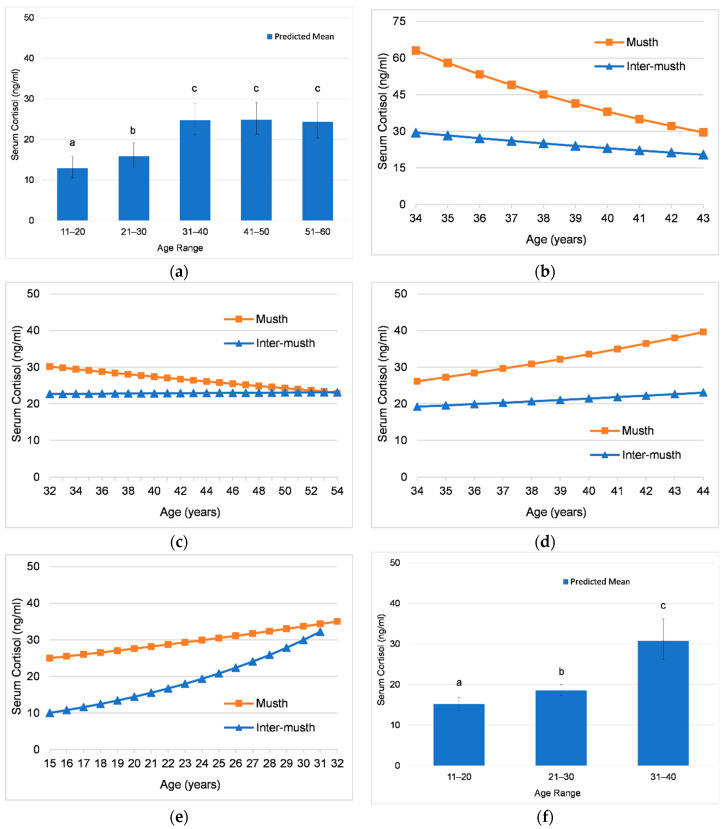
Age as a predictor of adrenal GC activity. Predictions from GLMMs for serum cortisol concentration comparing mean values across age categories (error bars represent standard error of the prediction) (**a**,**f**), and interactions of age and musth state showing how cortisol during musth and inter-musth changes with age (**b**–**e**). Different letters denote significant differences in mean values between age categories. (**a**) All elephants: Comparing mean values across age bins. (**b**) Male1: Interaction of age and musth state. (**c**) Male2: Interaction of age and musth state. (**d**) Male3: Interaction of age and musth state. (**e**) Male4: Interaction of age and musth state. (**f**) Male4: Comparing mean values across age bins.

There was an overall increase in mean serum cortisol concentrations with age in all bulls combined. Across bulls, mean cortisol concentrations were lowest in the 11–20-year age category, higher in ages 21–30 years, and highest in ages 31–60 years (Figure 4a). The change in cortisol over time varied within individuals; two bulls (Male3 and Male4) exhibited an increase in cortisol over time, one (Male1) exhibited a decrease, and in one (Male2), there was no significant change with increasing age (Table 11).

The interaction of age and musth (Figure 4b–f) shows that cortisol concentrations changed with age during musth and inter-musth periods. For all individuals and all bulls combined, the interaction of age and musth state was significant (Table 11). The rate of change in cortisol was greater during musth than inter-musth in Male1 (Figure 4b), Male2 (Figure 4c), and Male3 (Figure 4d), with relatively little change in inter-musth concentrations. By contrast, Male4 (Figure 4e) showed a higher rate of increase with age in inter-musth periods compared to musth periods, indicating that inter-musth concentrations increased more dramatically over time.

#### 3.2.3. Effects of Origin of Birth on Cortisol

When comparing cortisol concentrations between wild-born (*n* = 2) and zoo-born (*n* = 2) males, the youngest and oldest age categories each comprised only one individual, and origin was confounded by age; therefore, this comparison was limited to the 31–50-year age category (*N* = 1861 samples). There was no difference in mean serum cortisol concentrations between wild-born and zoo-born males (GLMM coefficient = −0.031, SE = 0.068, χ^2^ = 0.211, df = 1, *p* = 0.646).

#### 3.2.4. Effects of Social Life Events on Cortisol

During the study, one bull was transferred in from another facility (Male3), one bull died (Male1), and the other three were humanely euthanized (Male2, Male3, Male4). Collectively, these bulls experienced seven social life events involving other bulls; Male2 experienced four events (one transfer, three deaths); Male3 experienced one event (one death—Male4); and Male4 experienced two events (one transfer, one death—Male1). The transfer in of a bull had no significant effect on mean cortisol concentrations in either of the resident bulls (Male2 or Male4). Death of a bull was a significant predictor of mean cortisol concentration (GLMM coefficient = −0.427, SE = 0.129, χ^2^ = 11.017, df = 1, *p* = 0.001) in only one case (Male4 in response to the death of Male1), with cortisol being lower in the 60-day post-event time period. No other bulls responded to the death of another bull with a change in mean cortisol concentration.

#### 3.2.5. Effects of Mycobacterium Tuberculosis Detection and Treatment on Cortisol

In the three bulls diagnosed and treated for *Mycobacterium tuberculosis* (*M. tb*), mean cortisol concentrations were compared between time periods preceding detection of *M. tb* by positive TW culture and time periods before and after the start anti-TB therapy. In one bull, it was also possible to compare cortisol before and after the end of anti-TB therapy.

In Male3 and Male2, the period of transition from the last culture-negative TW to detection of active *M. tb* was 9 weeks and 10 weeks, respectively. Therefore, comparisons of cortisol concentrations were made between 30-day time blocks in the 120 days preceding *M. tb* detection. In the first bull diagnosed, Male4, the period of transition from the last culture-negative TW to detection of active *M. tb* was 14 months. Seroconversion also occurred within that time period, so comparisons in cortisol concentrations were made between this year-long transition period to the year prior, in addition to comparisons between 30-day time blocks in the 120 days preceding detection.

Detection of active TB was a significant predictor of mean cortisol concentration (GLMM coefficient = 0.346, SE = 0.146, χ^2^ = 5.607, df = 1, *p* = 0.019) in only one individual, Male3, with cortisol being higher in the 31–60-day time period prior to detection compared to the 61–90 and 91–120-day time periods prior to detection. Neither Male4 nor Male2 showed significant changes in mean cortisol concentrations with the transition to active TB. The start of anti-TB therapy was a significant predictor of mean cortisol concentration (GLMM coefficient = 0.222, SE = 0.122, χ^2^ = 3.331, df = 1, *p* = 0.048) only in Male3, with higher cortisol in the 30 days after starting therapy. Male2 was the only bull not undergoing anti-TB therapy at the time of euthanasia, and mean cortisol concentration was lower in the 30 days after therapy ended (GLMM coefficient = −0.354, SE = 0.133, χ^2^ = 7.046, df = 1, *p* = 0.008).

#### 3.2.6. Effects of Health Decline on Cortisol

The bull that died of sepsis (Male1) showed higher mean cortisol concentrations (GLMM coefficient = 0.548, SE = 0.211, χ^2^ = 6.753, df = 1, *p* = 0.009) in the final 30 days of life, with a sharp increase in the 4 days prior to death. In the three males that were euthanized, there were no significant differences in mean cortisol between the 30-day time period preceding euthanasia and the previous 30 days.

## 4. Discussion

This study confirmed coincident secretory patterns of cortisol and testosterone during musth in zoo-housed Asian elephant bulls. When assessing the impact of extrinsic factors (e.g., social or environmental changes) on adrenal GC activity, it is important to account for intrinsic GC secretion patterns, such as those associated with musth. Testosterone decreased with age in all adult bulls, whereas age-related patterns in cortisol varied across individuals. Acute adrenal responses in association with anti-TB therapy were exhibited in two bulls but were not clearly associated with transition to active TB. Except for a significant end of life cortisol increase in one bull, there was no clear evidence of prolonged elevated cortisol secretion outside of musth in any individual. In contrast to influences of physiological change related to musth cycles, there was no evidence of social changes (addition or removal of bulls) impacting adrenal GC activity in these bulls in the short term.

### 4.1. Longitudinal Serum Testosterone Patterns

#### 4.1.1. Musth Patterns

The bulls in this study exhibited regular annual musth cycles, each occurring at different times of year with no evidence of synchronization, but with varying musth durations and more overlap in some years such that multiple bulls were in musth at the same time, similar to patterns observed in wild bulls (reviewed in LaDue et al. [56]). There were no extended periods of elevated testosterone outside of musth; however, all bulls experienced testosterone elevations of short duration that in some cases also coincided with behavioral and physical correlates (i.e., aggression, TGS/UD) of musth. These episodic elevations were associated with a recurrence of musth, with musth cycles of other bulls, and presence of estrous females and/or breeding activity. Brief elevations in testosterone associated with presence of estrous females and mating were also found in previous studies [57,58,59]. Although the elephants in this study were fairly consistent in their annual musth cycles for much of their lives, variability both within and across individuals highlights the need to closely and consistently monitor each individual from an early age and throughout musth and inter-musth periods to determine the pattern for each bull to ensure proper and safe management.

Contrary to the expression of annual musth in this study, other studies [16,60,61] have shown a large proportion of adult males in captivity experience musth more than once a year, and age of the individual or presence of other males is not a reliable indicator of musth duration or frequency. Furthermore, not all bulls of adequate age exhibit musth in North American zoos [16,27]. In addition to the housing of multiple adult bulls, perhaps the age structure of the bulls at Oregon Zoo was a factor influencing musth expression on an annual basis similar to that observed in the wild. Keerthipriya and Vidya [62] found in wild Asian elephants that the presence of males in the oldest age class (45+ in that study) positively affected the overall occurrence of musth. Taking this a step further, Hartley et al. [42] suggested more broad considerations for social management of male elephants to better provide for their social needs across their life span, such as providing young male elephants the opportunity to associate with peers outside their family group or with temporary all-male groups to develop a range of social skills, or to spend time in a breeding herd with adult bulls to gain experience with reproductive interactions.

#### 4.1.2. Musth Threshold Testosterone Concentrations

Differences in testosterone corresponding to the beginning and end of musth suggest that threshold concentrations are individualistic. Although the minimum concentration defines the absolute threshold, the median and mean represented more typical concentrations defining full musth and were closer to the 50 ng/mL threshold found by Brown et al. [16] in captive Asian and African bulls. Furthermore, the minimum value for the first musth point defined by Chave et al. [27] corresponded more closely to the mean than the minimum in this study, suggesting the mean is a more appropriate measure of musth threshold concentrations.

#### 4.1.3. Effects of Age and Dominance Status on Testosterone across Bulls

Higher overall testosterone concentrations with social rank have been reported in captive [4,14] and wild [9] elephants; however, there were no definitive relationships between higher mean or musth threshold testosterone concentrations and age or dominance rank among Oregon Zoo bulls. This is in contrast to age-related increases in mean testosterone found in epidemiological studies [16,27]. However, these studies included males that had not exhibited musth, and a larger number of older males exhibited musth compared to younger males, so the age-related increase is in part due to the differential expression of musth. Thongtip et al. [63] found age-related decreases in circulating and seminal testosterone across Asian elephant bulls during inter-musth periods, with testosterone being highest in the 10–19-year age group, followed by the 23–43-year age group, and then lowest in the 51–70-year age group.

#### 4.1.4. Effects of Age on Testosterone within Individuals

Testosterone decreased with age overall and across age categories in all sexually mature bulls. The youngest male showed increased testosterone with age in the 5 years after his first musth cycle, and then a gradual decrease. In younger males, expression of musth is shorter and more sporadic, becoming more robust with increasing age [4,6,10,11]. All older bulls showed decreased mean testosterone past 31–40 years of age, providing evidence of age-related decreases in testosterone similar to those found in male model species [64] and humans [65,66].

### 4.2. Longitudinal Serum Cortisol Patterns

#### 4.2.1. Effects of Puberty, Musth, and Testosterone on Cortisol

Serum cortisol covaried positively with testosterone and musth as observed in previous studies [16,27,28]. Likely in part due to this positive correlation, sexual maturation was a significant predictor of mean cortisol concentration with cortisol being higher after sexual maturity. While the significance of increased adrenal GC activity during musth has not been determined, Yon et al. [28] provided evidence that the adrenal glands increase steroid production in general during musth, as measured by increases in cortisol, dehydroepiandrosterone (DHEA), and androstenediol. Explanations for the increase in cortisol during musth include the possibility that the state of musth is energetically costly and presents a physiological challenge [28,46]. It also may be associated with ecological stress, and increased secretory activity may help animals cope with these challenges. Cortisol may also increase due to high metabolic demand and reduced food intake, as found in other mammalian species [67].

It should be noted that findings of increased circulating cortisol during musth in our study and others 16,27,28] contrast with studies showing excreted fecal GC metabolites (FGMs) are not elevated during musth in captive and free-ranging African [43,68,69,70] and free-ranging Asian [71] elephant bulls. Kumar et al. [72] showed increased FGMs during musth in captive Asian bulls but suggested the increase might be due to musth management practices rather than the physiological state of musth. One obvious difference between these studies is the measurement of circulating versus excreted GCs. From a metabolic perspective, it seems plausible that metabolic changes that occur during musth [46] could influence concentrations in excreted steroids. Bulls in musth may spend less time feeding, and zoos report voluntary food reduction, inappetence, and weight loss [46]. Changes in food availability have been shown to trigger large fluctuations in the metabolic rate of free-living animals [73]. It is also possible this could affect fecal concentrations as there is no reliable method for indexing steroid concentrations to account for fluctuations in food intake. In other ungulates, the analogous condition of rut has been shown to be a metabolically challenging period for males with active selection for those in good condition [74], and rut has been associated with increases in fecal GC metabolites [75]. Metrione et al. [76] observed lower FGMs in bison during winter months when their metabolic rate was also low, suggesting a metabolic strategy in which GCs are minimally secreted in order to conserve energy when forage is scarce. Finally, with the amount of increase in circulating testosterone being much larger than amount of increase in cortisol during musth, it seems possible that any “dampening” of the pattern as a result of metabolic changes could result in negligible change in GC metabolites while still maintaining a rise in testosterone. Clearly, further studies are needed to elucidate how adrenal GC activity is altered in relation to metabolic changes and the physiological changes associated with musth.

#### 4.2.2. Effects of Age on Cortisol across Bulls

Overall, mean cortisol concentrations increased with age with all adult bulls combined, being lowest in the 11–20-year age group, which included early musth cycles in the youngest bull, higher in 21–30-year age group, and highest in ages 31–60 years. This overall increase with respect to age across bulls is in agreement with previous studies [16,27].

#### 4.2.3. Effects of Age on Cortisol within Individuals

Although cortisol covaried with testosterone and increased during musth in all bulls, age-related changes in cortisol concentrations indicated that the amount of cortisol increase during musth was changing over time. Age-related changes in cortisol concentrations varied across individuals, with an increase in cortisol over time in two bulls, a decrease in one bull, and no significant change in one bull.

The interaction between age and musth state was significant in all bulls, indicating cortisol concentrations changed differentially during musth versus inter-musth periods with age. The three older bulls exhibited the greatest magnitude of change during musth, with much smaller changes in inter-musth concentrations. However, the pattern of change differed across bulls, which could be related to social factors, musth, or simply individual differences. Male1 and Male3 were the same age during the study; Male1 showed cortisol concentrations during musth decreased over time, and he was with females only infrequently for breeding and only in the first half of the study, whereas Male3 showed cortisol concentrations that increased during musth over time, and he was breeding and integrated with the female herd with frequent social opportunities. Interestingly, Male2 showed a narrower range in cortisol concentrations between musth and inter-musth periods than other bulls, which narrowed further over time such that mean concentrations did not differ between musth and inter-musth periods. It is plausible that this merging of musth and inter-musth cortisol values could be related to Male2′s erratic musth cycles in the last few years of his life. The role of cortisol during musth needs further investigation to explore age-related differences and whether any changes could be related to social dynamics.

The youngest male exhibited a higher rate of change in inter-musth cortisol concentrations, with an increase of approximately 20 ng/mL, which could be related to exacerbation of his joint issues similar to other cases of poor joint health in elephants [77,78]. In the three older bulls, inter-musth basal cortisol levels did not increase with age, suggesting these individuals maintained good body condition and healthy metabolic profiles, consistent with findings by Norkaew et al. [79] for Asian elephant bulls ages 16–50 in tourist facilities in Thailand.

#### 4.2.4. Effects of Origin of Birth on Cortisol

Mean cortisol concentrations did not differ significantly between wild-born and zoo-born males with the comparison limited to the 31–50 age group. However, another important factor is life history, and the wild-born males in this study (Male1 and Male3) experienced multiple inter-zoo transfers, whereas one zoo-born male (Male4) experienced only two transfers and the other (Male2) spent his entire life at the Oregon Zoo. In other species, cortisol secretion later in life can be influenced by early life trauma [80] or early social experience influencing development of the stress response [81,82]. Prado-Oviedo, et al. [83] reported differences in the early lives of wild-born versus captive-born elephants that could have long-term welfare implications, and Greco et al. [84] suggests that factors such as travel, unfamiliar surroundings, and social separation may explain higher rates of stereotypic behavior associated with number of transfers.

#### 4.2.5. Effects of Social Life Events on Cortisol

Social changes with the addition of a new adult bull and deaths of other bulls had minimal impact on adrenal response in resident males, with no significant changes in cortisol associated with the transfer and only one male exhibiting a change in cortisol following a death. Although housed separately from each other, bulls did have olfactory and acoustic contact, and it was presumed they had hormonal influences on each other, which was supported by testosterone increases of a short duration that coincided with other bulls in musth. Social factors can influence musth expression in bulls [16]. Perhaps if social factors had presented significant psychological stressors leading to chronic GC elevation for any individual, then we might expect that individual to show a short-term decrease in cortisol with the death of another bull depending on dominance rank. However, there was no evidence of chronically elevated or depressed GC levels in these bulls.

#### 4.2.6. Effects of Mycobacterium Tuberculosis Detection and Treatment on Cortisol

Although cortisol has been shown to increase in association with pathologies such as gastrointestinal issues, skin lesions, lameness, foot lesions [77], an increase associated with activation of *M. tb* disease was observed in only one individual, suggesting cortisol is not a good biomarker for identifying an immune response triggered by cytokines [85] or other factors in the transition from latent to active TB.

The increase in cortisol after starting anti-TB therapy in one individual, and the decrease after ending therapy, supports caretaker accounts that the onset of and recovery from adverse treatments side effects [86] were quick. These adrenal responses suggest that GC monitoring may provide an additional tool for assessing well-being during certain medical treatments. In Male2, age-related changes in musth characteristics made treatment with oral medications even more challenging. One of his typical musth symptoms was inappetence. Therefore, treatment became more difficult when his musth episodes became more erratic and he was showing musth signs more frequently. Elephant care staff developed a feeding regimen to manage the intensity of his musth, allowing him to exhibit only a “mild” musth, which allowed staff to continue to administer oral medication and ensured cooperation for therapy.

### 4.3. Future Directions

These cortisol data provided no evidence of chronic stress, but did show elevations in some cases involving medical treatments and health declines associated with physical discomfort. Thus, it might be beneficial to include other measures of stress/well-being, such as immunoglobulin-A (IgA) to help inform health and “end of life” decision making. IgA is an immune protein associated with pathogen defense, and has been demonstrated to downregulate during times of stress and increase in response to positive stimuli (reviewed in Edwards et al. [47]). Including IgA as a biomarker could contribute to a more rounded assessment for welfare that considers both positive and negative perspectives. Age-related changes in dehydroepiandrosterone (DHEA), an adrenal hormone shown to increase in musth [28] and decrease with advanced age in humans [87], could also provide insight into the relationship between adrenal GC activity and metabolic changes, and aid in the care of geriatric elephants.

## 5. Conclusions

This longitudinal study contributes to our knowledge of individual variabilty in bull reproductive physiology, which can help support the sustainability of managed elephant populations. Musth is a condition that can create both human safety and animal welfare concerns. It is therefore paramount, for improved bull management, to understand the physiological changes associated with musth, to monitor musth characteristics on an individual basis and from an early age, and to have tools available for assessing the well-being of male elephants of all ages and all health conditions. Findings from this study have implications for how we manage male elephants, but also emphasize the need for further investigations into the role of GCs in the physiology of musth and the importance of taking intrinsic patterns into account when assessing adrenal response to external stimuli (e.g., social or environmental changes) or to physical or physiological changes (e.g., aging, health decline). Cortisol has been shown to increase with end-of-life health declines in some individuals [29], and this was the case in the one bull that died of sepsis, but not in the two bulls that were euthanized due to joint pathologies or in the fourth bull that was euthanized due to development of a drug-resistant strain of *M. tb* but was not exhibiting signs of health decline [51]. The differential responses may in part be due to the point in decline at which the euthanasia decision was made, or to the nature of the health decline itself.

Results from this study will be useful in ongoing research investigating immune function during musth. Testosterone has been shown in other species to have immunosuppressive effects [88]. It is therefore possible that the occurrence of musth could be associated with immunosuppression in bull elephants, which, combined with a decline in food intake and behavior changes, could have important implications for TB management in bull elephants. Lastly, we continue to learn from these bulls, as this dataset and banked serum will be part of ongoing investigations to identify additional biomarkers, such as those associated with inflammation and immune function, that can be used for assessing how environmental and physiological factors affect animal health over time.

## Figures and Tables

**Table 1:** Male elephants included in this study. Individual, origin, age range during study, whether the individual exhibited normal musth cycles, number of samples analyzed, and statistical analyses performed for each individual.

Elephant	Origin	Birth Year	Age Range (Years)	Offspring Sired	Effects Analyzed
Male1	Wild	1960	~34–43	4	M, Age, DS
Male2	Zoo-born Oregon Zoo	1962	32–54	7	M, Age, TM, DM, Tbactive, TBtr, DS
Male3	Wild	1971	~34–44	6	M, Age, DM, Tbactive, TBtr, DS
Male4	Zoo-born Oregon Zoo	1983	12–32	0	R, M, Age, TM, DM, Tbactive, TBtr, DS

R = reproductive state; M = musth hormone concentrations; Age = age analysis; TM = transfer in of another adult male; DM = death of another adult male; TBactive = conversion to active TB; TBtr = anti-TB therapy; DS = health decline leading to death or euthanasia.

**Table 2:** Temporal gland swelling and secretion (TGS).

Scale	Definition	Pictoral Definition	
0	No visible swelling or secretion.	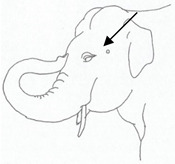	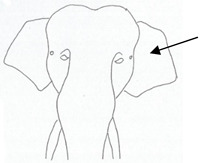
1	Swollen temporal gland area. Opening may be enlarged. May not be symmetrical.	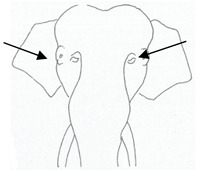	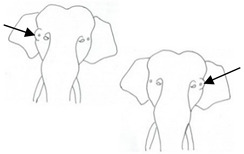
2	Temporal gland area wet. TGS less than 1/4 way to jawline.	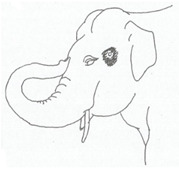	
3	TGS between 1/4 and 3/4 way to jawline.	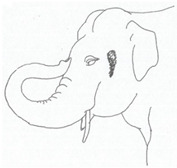	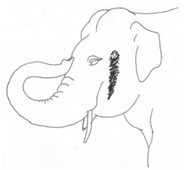
4	TGS from 3/4 to all the way to jawline.	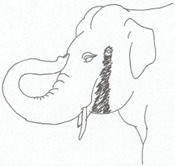	
5	TGS staining is dried (lighter color than wet).	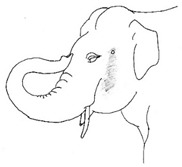	

**Table 3:** Urine dribbling (UD).

Scale	Definition	Pictoral Definition	
0	No visible UD. Urination occurs normally (with penis fully extended).	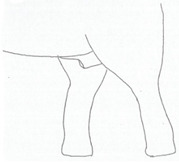	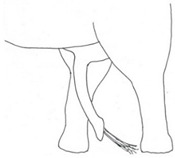
1	Occasional drops (without penis extended). Urination occurs with penis partially extended.	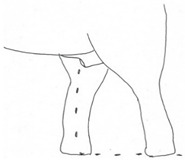	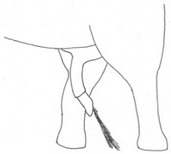
2	Regular drops and/or some steady streams (without penis extended). Urination occurs with penis less extended less than #1. Legs (upper or lower) a little wet or stained with urine.	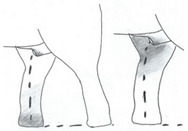	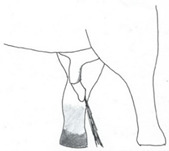
3	Steady streams from the opening and some dribbling from the skin of the sheath (without penis extended). Penis does not drop to urinate. Legs (upper or lower) half wet or stained with urine. Skin around sheath wet with urine.	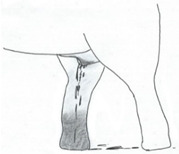	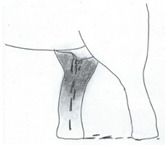
4	Heavy steady streams falling from the opening and skin of the sheath so the stream looks wider than #3 (without penis extended). Penis does not drop to urinate. Legs entirely wet with urine. Skin around sheath wet with urine.	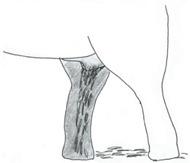	
5	UD staining is dried (lighter color than wet).	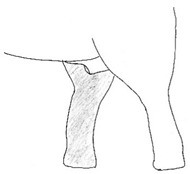	

**Table 4:** Musth behavior scale for Oregon Zoo male elephants.

Scale	Definition
0	No aggression, moves readily
1	Somewhat “spacey” or uncooperative
2	Reluctant to move, bangs on doors occasionally
3	Considerable hesitation moving, bangs on doors often
4	Very aggressive, refuses to move

**Table 5:** Criteria used to define musth in this study.

Musth Status	Serum Testosterone		TGS ^a^	UD ^b^	Behavior ^c^
	Concentration	Duration			
Inter-musth and Non-musth	<10 ng/mL	>2 weeks	0 ^d^	0	0 ^d^
Pre-musth	>10 ng/mL	no criteria	1	0	1–4
Musth	>10 ng/mL	>4 weeks (including pre/post musth)	2–4	1–4	1–4
Post-musth	>10 ng/mL	no criteria	5 ^e^	5 ^e^	1–4

^a^ Numerical values defined in Table 2. ^b^ Numerical values defined in Table 3. ^c^ Numerical values defined in Table 4. ^d^ Behaviors typical of musth and mild TGS were sometimes observed during inter-musth periods coincident with musth in other bulls, estrous in females, or breeding activity with females. ^e^ TGS/UD of 5 (versus 0) indicates cessation of TGS and UD but with signs of recent occurrence.

**Table 6:** Serum testosterone concentration (ng/mL) for males across all reproductive states and during periods of musth and inter-musth. Individual, birth origin, age range during study, number of samples measured for serum testosterone, concentration median, range, mean, standard deviation (SD), and coefficient of variation (CV).

				Overall			Inter-Musth Period ^a^			Musth Period		
Individual	Origin of Birth	Age Range (Years)	*N*	Median (Range)	Mean (SD)	CV%	Median (Range)	Mean (SD)	CV%	Median (Range)	Mean (SD)	CV%
Male1	Wild	~34–43	350	5.07 (0.10–258.88)	29.49 (44.45)	150.74	1.98 (0.10–103.42)	5.31 (11.44)	215.99	82.10 (0.55–258.88)	79.53 (45.65)	57.39
Male2	Zoo-born	32–54	1087	7.25 (0.10–300.26)	21.19 (33.66)	158.83	2.89 (0.10–120.18)	6.35 (10.46)	164.68	38.47 (0.10–300.26)	49.64 (44.16)	88.95
		34–44 ^a^	485 ^b^	8.70 (0.10–300.26)	28.79 (41.73)	144.94	3.68 (0.10–120.18)	7.68 (12.08)	157.28	48.53 (0.12–300.26)	59.39 (49.76)	83.79
Male3	Wild	~34–44	468	2.40 (0.10–99.10)	9.76 (14.14)	145.23	0.51 (0.10–43.17)	1.92 (4.25)	221.58	17.23 (0.10–99.10)	21.31 (16.20)	76.04
Male4 ^b^	Zoo-born	12–32	939	4.60 (0.10–141.65)	15.24 (23.51)	154.32	0.69 (0.10–76.17)	3.41 (6.65)	194.96	36.72 (0.10–141.65)	41.58 (28.57)	68.72
		11–15 ^c^	179	4.13 (0.10–56.57)	5.97 (6.76)	113.12	-	-	-	-	-	-
		15–32 ^d^	760	4.67 (0.10–141.65)	17.24 (26.29)	146.09	0.69 (0.10–76.17)	3.41 (6.65)	194.96	36.72 (0.10–141.65)	41.58 (28.57)	68.72
All Elephants		~12–54	2734	5.12 (0.10–300.26)	18.25 (30.26)	165.78	1.34 (0.10–120.18)	4.56 (8.97)	196.70	34.96 (0.10–300.26)	46.02 (39.68)	86.23

^a^ Age range limited for comparisons to Male1 and Male3 at similar chronological ages. ^b^ Inter-musth period includes only data between musth periods and does not include data prior to the first occurrence of musth. ^c^ Age range limited to time period prior to first musth at age 15. ^d^ Age range limited to time period after first musth at age 15.

**Table 7:** Serum testosterone concentrations (ng/mL) corresponding to the beginning and end of musth.

Individual	Threshold Testosterone Concentration (ng/mL)			Testosterone Concentration for First Musth Point Using Methods of Chave et al., 2019 [27]
	Minimum	Median	Mean	
Male1	16	69	72	67
Male2	11	43	49	51
Male3	13	16	18	21
Male4	10	40	39	35

**Table 8:** Age as a predictor of testosterone. Individual, age range of analysis, age variable (age, age category), effect size with standard error (SE), Wald statistic, and *p*-value from GLMMs; and relative effect of age on mean testosterone concentrations. Degrees of freedom (df) is 1 in all pair-wise comparisons.

Individual	Age Range of Analysis	Age Variable	Effect Size (SE)	Wald	*p*	Age Effect
Male1	~34–43	Age (years)	−0.066 (0.021)	9.681	0.002	Decreasing
		31–40 (reference) ^a^				
		41–50	−0.097 (0.125)	0.606	0.436	-
Male2	32–53	Age (years)	−0.027 (0.004)	37.043	<0.001	Decreasing
		31–40 (reference) ^a^				
		41–50	−0.289 (0.056)	26.262	<0.001	Lower
		51–60	−0.432 (0.088)	23.858	<0.001	Lower
Male3	~34–44	Age (years)	−0.090 (0.015)	38.079	<0.001	Decreasing
		31–40 (reference) ^a^				
		41–50	−0.495 (0.082)	36.783	<0.001	Lower
Male4 ^b^	15–32	Age (years)	−0.017 (0.005)	10.509	0.001	Decreasing
		11–20 (reference) ^a^				
		21–30	−0.081 (0.063)	1.655	0.198	-
		31–40	0.167 (0.128)	1.697	0.193	-

^a^ Reference category for GLMMs against which other age categories are compared. ^b^ Age analysis includes only mature bulls, which started at age 15 for this individual.

**Table 9:** Serum cortisol concentration (ng/mL) for males across all reproductive states and during periods of musth and inter-musth. Individual, birth origin, age range during study, number of samples measured for serum cortisol, and concentration median, range, mean, standard deviation (SD), and coefficient of variation (CV).

				All Reproductive States			Inter-Musth Period			Musth Period		
Individual	Origin of Birth	Age Range (Years)	*N*	Median (Range)	Mean (SD)	CV%	Median (Range)	Mean (SD)	CV%	Median (Range)	Mean (SD)	CV%
Male1	Wild	~34–43	380	27.70 (5.40–261.60)	36.68 (26.51)	72.29	23.30 (5.40–261.60)	30.28 (25.28)	83.48	44.40 (15.20–125.80)	49.90 (23.77)	47.63
Male2	Zoo-born	32–54	1099	23.50 (4.40–141.30)	28.19 (17.21)	61.06	21.55 (4.40–141.30)	27.10 (17.78)	65.60	26.95 (5.00–96.00)	30.18 (16.16)	53.53
Male3	Wild	~34–44	525	24.80 (2.50–109.70)	29.92 (19.87)	66.39	19.90 (2.50–106.10)	24.82 (15.91)	64.12	32.50 (6.50–109.70)	38.04 (22.24)	58.48
Male4 ^a^	Zoo-born	12–32	934	22.30 (2.50–176.30)	27.23 (20.08)	73.75	18.50 (2.50–176.30)	24.72 (20.66)	83.59	33.80 (4.80–128.30)	34.78 (18.64)	53.59
All Elephants		~12–54	2938	24.00 (2.50–261.60)	29.29 (20.22)	69.03	24.00 (2.50–261.60)	26.54 (19.74)	74.40	30.75 (4.80–128.30)	35.65 (20.26)	56.85
Wild-born		~34–44	905	26.20 (2.50–261.60)	32.76 (23.12)	70.58	21.85 (2.50–261.60)	27.39 (21.01)	76.71	37.15 (6.50–125.80)	43.08 (23.67)	59.54
Zoo-born		12–54	2033	23.10 (2.50–176.30)	27.75 (18.59)	66.98	20.90 (2.50–176.30)	26.10 (19.06)	73.04	28.90 (4.80–128.30)	32.14 (17.39)	54.11

^a^ Age range limited for comparisons to Male1 and Male3 at similar chronological ages.

**Table 10:** Testosterone and musth as a predictor of adrenal glucocorticoid (GC) activity. Fixed effect, effect size with standard error (SE), Wald statistic, *p*-value from GLMMs, and relative effect of testosterone and musth state on mean cortisol concentrations. Degrees of freedom (df) is 1 in all pair-wise comparisons.

Variable	Individual(s)	Effect Size (SE)	Wald	*p*	Effect on Mean Cortisol
Testosterone	Male1	0.129 (0.014)	85.048	<0.001	Positive
	Male2	0.028 (0.009)	8.504	0.004	Positive
	Male3	0.128 (0.013)	61.284	<0.001	Positive
	Male4	0.070 (0.011)	38.353	<0.001	Positive
	All Elephants	0.075 (0.006)	161.041	<0.001	Positive
Inter-musth (reference) ^a^					
Musth	Male1	0.259 (0.027)	93.600	<0.001	Higher
	Male2	0.060 (0.016)	14.523	<0.001	Higher
	Male3	0.187 (0.024)	61.284	<0.001	Higher
	Male4	0.204 (0.024)	74.687	<0.001	Higher
	All Elephants	0.152 (0.011)	194.919	<0.001	Higher

^a^ Reference category for GLMMs.

**Table 11:** Age as a predictor of adrenal GC activity. Individual, age range of analysis, age variable (age, age category, interaction of age and cycle), effect size with standard error (SE), Wald statistic, *p*-value from GLMMs, and relative effect of age on mean cortisol concentrations. Degrees of freedom (df) is 1 in all pair-wise comparisons.

Individual	Age Range of Analysis	Age Variable	Effect Size (SE)	Wald	*p*	Age Effect
Male1	~34–43	Age (years)	−0.026 (0.005)	32.714	<0.001	Decreasing
		31–40 (reference) ^b^				
		41–50	−0.110 (0.029)	14.189	<0.001	Lower
		Interaction of Age and Musth	−0.019 (0.009)	4.346	0.037	Significant
Male2	32–54	Age (years)	−0.002 (0.001)	2.079	0.149	--
		31–40 (reference) ^b^				
		41–50	−0.028 (0.017)	2.714	0.099	--
		51–60	−0.022 (0.021)	1.092	0.296	--
		Interaction of Age and Musth	−0.006 (0.002)	5.605	0.018	Significant
Male3	~34–44	Age (years)	0.011 (0.004)	8.054	0.021	Increasing
		31–40 (reference) ^b^				
		41–50	0.113 (0.023)	24.856	<0.001	Higher
		Interaction of Age and Musth	0.010 (0.008)	1.451	0.228	--
Male4 ^a^	15–32	Age (years)	0.025 (0.002)	118.624	<0.001	Increasing
		11–20 (reference) ^b^				
		21–30	0.087 (0.026)	11.171	0.001	Higher
		31–40	0.306 (0.042)	53.358	<0.001	Higher
		Interaction of Age and Musth	−0.023 (0.005)	22.151	<0.001	Significant
All Elephants	15–54	Age (years)	0.004 (0.011)	19.502	<0.001	Increasing
		11–20 (reference) ^b^				
		21–30	0.090 (0.023)	15.450	<0.001	Higher
		31–40	0.283 (0.035)	65.123	<0.001	Higher
		41–50	0.284 (0.037)	58.378	<0.001	Higher
		51–60	0.276 (0.041)	44.934	<0.001	Higher
		Interaction of Age and Musth	−0.007 (0.001)	42.213	<0.001	Significant

-- No significant difference or interaction is not significant. ^a^ Age analysis includes only mature bulls, which started at age 15 for this individual. ^b^ Reference category for GLMMs.

## Data Availability

Data restrictions apply to the de-identified data underlying the findings of this study to protect the facilities and animals included in this study from potential abuse of this information. Data can be made available upon request to researchers who meet the criteria for access to confidential data, with approvals from registrars at the Oregon Zoo (peter.grimm@oregonzoo.org).
